# Dual learning processes underlying human decision-making in reversal learning tasks: functional significance and evidence from the model fit to human behavior

**DOI:** 10.3389/fpsyg.2014.00871

**Published:** 2014-08-12

**Authors:** Yu Bai, Kentaro Katahira, Hideki Ohira

**Affiliations:** Department of Psychology, Graduate School of Environmental Studies, Nagoya UniversityNagoya, Japan

**Keywords:** reinforcement learning model, reversal learning, learning rate, decision making, value

## Abstract

Humans are capable of correcting their actions based on actions performed in the past, and this ability enables them to adapt to a changing environment. The computational field of reinforcement learning (RL) has provided a powerful explanation for understanding such processes. Recently, the dual learning system, modeled as a hybrid model that incorporates value update based on reward-prediction error and learning rate modulation based on the surprise signal, has gained attention as a model for explaining various neural signals. However, the functional significance of the hybrid model has not been established. In the present study, we used computer simulation in a reversal learning task to address functional significance in a probabilistic reversal learning task. The hybrid model was found to perform better than the standard RL model in a large parameter setting. These results suggest that the hybrid model is more robust against the mistuning of parameters compared with the standard RL model when decision-makers continue to learn stimulus-reward contingencies, which can create abrupt changes. The parameter fitting results also indicated that the hybrid model fit better than the standard RL model for more than 50% of the participants, which suggests that the hybrid model has more explanatory power for the behavioral data than the standard RL model.

## Introduction

One of the fundamental questions in a study on decision making is how animals and humans select actions in the face of reward and punishment and how they continually update their behavioral strategies according to changes in the environment. The theories of reinforcement learning (RL) have provided a powerful theoretical framework for understanding such processes (Sutton and Barto, 1998). RL theories postulate that actions are chosen to maximize expected rewards based on value functions, which are subjective estimates of future rewards. The value functions are continually updated based on the reward prediction error, which is the mismatch between the expected and actual rewards. Based on this concept, the prediction error quantified in the theories of conditioning, such as the Rescorla-Wagner (RW) model (Rescorla and Wagner, 1972), has helped to explain the choice behavior of humans and animals (Daw and Doya, 2006; Corrado and Doya, 2007). The RW model was originally proposed as a classical conditioning model in which the animal is assumed to learn the associative strength between cues and outcome from the prediction errors. This model uses the prediction errors to drive the change of associative strength. The association between the cues and outcome will be strengthened if the error is positive, whereas this association will weaken or might become negative if the error is negative. In the RL literature, the update rule of associative strength has been used to update the value function, which is used for action selection.

In contrast to the RW model, the Pearce and Hall (PH) model (Pearce and Hall, 1980) utilizes the prediction error to control the emphasis placed on the cues in subsequent trials, which indicate the amount of information that will be obtained from the cues. Large prediction errors will increase the amount of attention devoted to the cues that accompanied the errors, thereby facilitating subsequent learning. Small prediction errors will weaken the attention and hamper learning. In the PH model, “attention” is modeled as a learning rate; thus, the prediction errors are used to update the learning rate, whereas the R-W model used a constant learning rate.

According to a number of neuroscientific studies, although the dopaminergic system reports the reward prediction error (Schultz, 1997), the amygdala reports the attention signal that is modulated by the reward prediction error, as predicted by the PH model (Roesch et al., 2010; Li et al., 2011). Thus, the learning signals of the RW and PH models likely coexist in different regions of the brain. A hybrid model that incorporates updates in the reward-prediction error in the RW model and updates in the learning rate in the PH model were proposed and explained for neural activities in the striatum, which is a major target of dopaminergic projection and neural activities in the amygdala (Li et al., 2011). In the hybrid model, the quantity of value updating in a trial is determined by the product of the prediction error and the learning rate, the quantity of which can be controlled by a constant parameter, thereby enabling the decision maker's behavior to dynamically adapt to the task. In contrast to the hybrid model, the product of the prediction error and the constant learning rate in the RW model determine the amount of action value updating, and thus any action based on the RW model is inflexible to adapt to a change in the reinforcement contingencies in some cases. However, in the PH model, the amount of action value updating is determined by the product of the reward value itself and the learning rate, which only depends on the prediction error that occurred in the prior trial; therefore, the PH model would result in unstable behavior when the decision maker receives a punishment. The mechanism of the hybrid model is supported by some evidence from a different perspective. Several Bayesian approaches (Kakade and Dayan, 2002; Courville et al., 2006; Preuschoff and Bossaerts, 2007) suggested that after a surprising event, animals should pay more attention to stimuli and that these events provoke the animals into learning faster. These studies provided a normative interpretation for the mechanism that is assumed in hybrid models.

Although in a classical conditioning task, neural correlates of the learning signals assumed in the hybrid model have been found (Li et al., 2011), in a complicated instrumental learning task with human participants, the functional significance of the hybrid learning algorithm remains unexplored.

In the present study, we used computer simulations to investigate a functional role of the hybrid model in a probabilistic reversal learning task. The probability learning task is widely used across species to understand decision making mechanisms. In the probabilistic reversal learning task, a decision maker is required to have two types of conflicting abilities to maximize their reward. First, he/she must be able to discriminate the difference in rewards between the good and bad options as quickly as possible, i.e., he/she should be sensitive to the difference between the two options. This action includes the ability to switch preference when the good option changes. Second, once the decision maker detects the good option, he/she should choose that option consistently even if he/she occasionally receives punishment, i.e., he/she should be slightly insensitive to the difference between the two options. If the hybrid model can exactly describe the dynamic changing of these abilities in this complicated decision making task, the performance based on a hybrid model would be better than that based on a standard RL model in some cases. Additionally, in contrast to Li et al. (2011), which applied the hybrid model to a classical conditioning task that without an action selection, the present study provide a RL model for an instrumental learning task based on the hybrid model Thus, our model includes the action selection rule described in Equation 2. Furthermore, by using a statistical model fit to human behavioral data, we assessed whether the hybrid model provides a better explanation for human decision-making behavior compared with the standard RL algorithm based on the RW model.

## Computer simulations

### Methods

#### Reversal learning task

An alternative choice task, a reversal learning task, was used for the computer simulations. In this task, the decision makers are presented with two response options. Rewards and losses, which could be obtained for an option, were distributed probabilistically and varied in frequency. Rewards and losses were set independently of the decision makers' previous choice. After the decision makers learn to choose the correct response in multiple trials using action feedback, the action-outcome contingency is reserved without explicit instruction. At that point, the decision makers cannot receive the expected feedback, which was used in previous trials to cue the decision makers to adapt their internal representations to reflect the reversal. Therefore, reversal learning relies on the flexibility of the action switching for an alternative response when the prior action is no longer rewarding. The reversal learning task has been induced in a trial-by-trial probabilistic manner in various studies (Cools et al., 2002; O'Doherty et al., 2003).

The task comprised 160 trials. During the first 80 trials, one option is an advantageous option, in which the reward/loss frequency ratio was 70:30, whereas the other option is a disadvantageous option, in which the reward/loss frequency ratio was 30:70. The contingencies were reversed in the last 80 trials. To investigate the functional role of learning algorithms under a different task difficulty, we manipulated the reward/loss frequency ratio, which determines how difficult it is to distinguish a good choice, according to three levels (the reward/loss frequency ratios in the three levels are 80:20, 70:30, and 60:40; easier tasks have a higher ratio).

### Reinforcement learning models

RL involves learning predictions of the future reward that will be obtained by performing a particular action. Many different varieties of RL algorithms exist. In this study, we compared how a standard Q-learning model and hybrid model described decision makers' choices. A Q-learning model, which is a standard RL model, updates action values based on the RW model (Watkins and Dayan, 1992; Sutton and Barto, 1998). The Q-learning model represents the estimated action value of each action (selecting one option) as *Q*-values. Let *Q*_a(t)_(t) denote the *Q*-value for option a (*t*)(a(*t*) = 1,2) in trial *t*. The *Q*-values are updated according to the action and resulting outcome. Let a(*t*) (=1,2) denote the option chosen by the decision maker in trial *t*. the *Q*-value corresponding to the selected target is updated as follows:

(1) $$Q_{\left(\text{a}(t\;+\;1)\right)}(t+1)=Q_{\text{a}(t)}(t)+a\left(R(t)-Q_{\text{a}(t)}(t)\right),$$

where the *Q*-value corresponding to the unselected target does not change. Here, α is the learning rate that determines the degree of the update and *R*(*t*) is the reward value of the choice during trial *t*, which is equal to 1 if the reward is provided in trial t and is equal to zero if punishment is provided.

Given a *Q*-value set, a choice is made according to the soft-max function, where the probability of choosing option 1 is as follows:

(2) $$P(\text{a}(t)=1)=\frac{1}{1+\exp\left[-\beta \left(Q_{1}(t)-Q_{2}(t)\right)\right]},$$

where *P*(a(*t*) = 2) = 1 − P(a(*t*) = 1). Here, β is the degree of stochasticity in making the choice (i.e., the exploration/exploitation parameter). The hybrid model differs from the Q-learning model only in updating the rule of action values. Whereas Q-learning treats the learning rate as a constant, the hybrid model incorporates learning rate modulation, as proposed by Pearce and Hall (1980), as well as Rescorla-Wagner's error-driven value update rule. In the hybrid model, the *Q*-value corresponding to the selected option is updated as follows:

(3) $$Q_{\left(\text{a}(t\;+\;1)\right)}(t+1)\;=Q_{\left(\text{a}(t\;+\;1)\right)}(t)+\alpha (t)\left(R(t)-Q_{\left(\text{a}(t)\right)}(t)\right)\text{​},$$

(4) $$\alpha (t+1)\;=\eta \left|R(t)-Q_{\left(\text{a}(t)\right)}(t)\right|+(1-\eta )\alpha (t),$$

Trial t's learning rate α (t) depends on prediction errors of past trials but not that of the current trial. η is a constant parameter that controls the level of influence from past trials to the current learning rate. A choice is made based on a soft-max function, which is the same as in the Q-learning model (Equation 2).

We used computer simulations to compare the average rewards gained by the standard Q-learning model and hybrid model when performing the reversal learning task. In the simulations, for the hybrid model, three parameters were systematically varied as follows: (a) the initial learning rate: α_0_ (α(1) = α_0_), (b) the exploration/exploitation parameter: β, and (c) the level of influence of past trials to current learning rate: η. The range of the initial value of the learning rate α_0_ varies from 0 to 1 with a 0.05 step. The initial value of β ranges from 0 to 50 with a 1.0 step. The value of η ranges from 0 to 0.1 with a 0.01 step and from 0.1 to 1 with a 0.1 step. The hybrid model in which η = 0 corresponds to the standard Q-learning model. Thus, the standard Q-learning model only differed from the hybrid model in that there is no parameter η. The values of α_0_ and β have the same ranges as in the hybrid model.

### Results

**Figure 1:**
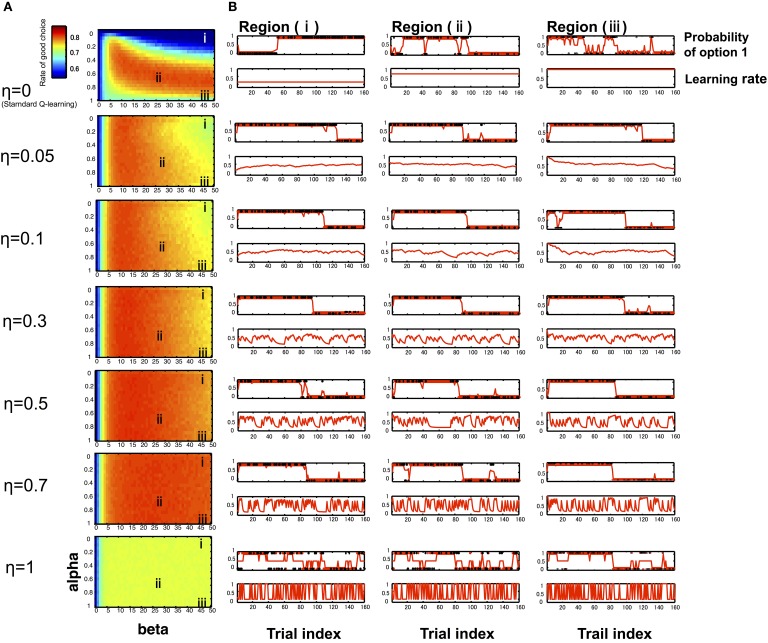
**Simulation results of RL models**. We used computer simulations to compare the Q-learning model and hybrid model performing a reversal learning task with 160 trials. To examine the model performance in various learning settings, simulations were repeated for varying initial learning rates α_0_ (0–1) and the exploration/exploitation parameter β (0–50) at different η levels (0–1). **(A)** With the rate of advantageous choice across all combinations of α_0_ and β at different η levels (seven typical levels: 0, 0.05, 0.1, 0.3, 0.5, 0.7, 1), particularly when η = 0, the model corresponds to the Q-learning model. Each cell depicts the proportion of advantageous choice, which was computed by simulating learning tasks 1000 times for each model. The resulting 2-dimensional plots were sufficiently smooth (Figure 1A), which suggested that the estimated average values were reliable. The initial learning rate α_0_ is varied on the y-axis, and the exploration/exploitation parameter β is varied on the x-axis. The region (i) indicates a mistuned parameter situation (α_0_ = 0.15; β = 45), the region (ii) indicates a good situation (α_0_ = 0.65; β = 25), and the region (i) indicates a mistuned parameter situation (α_0_ = 0.95; β = 45). **(B)** Typical time courses of the likelihood of choosing option 1 (the good choice before the reversal occurs) and the learning rate, with the same combination of α_0_ and β on the left-side panel. The learning rate curves illustrate that the learning rate changes frequently as the parameter η value increases. The likelihood of choosing option 1 curves indicates that the agent can detect the reversal of the good option faster as η value increases.

In the computer simulation, we compared the proportions of the advantageous choice of the standard Q-learning model and hybrid model. The results depicted in Figure 1 illustrate that there is not a substantial difference between the hybrid model and standard Q-learning model in a good parameter set, such as region (ii) (α_0_ = 0.65, β = 25). However, the hybrid model outperforms the standard Q-learning model (η = 0) in a mistuned parameter situation, e.g., when the learning rate is relatively low and the exploration/exploitation parameter is relatively high, such as region (i) (α_0_ = 0.15; β = 45) or the learning rate is relatively high and the exploration/exploitation parameter relatively high, such as region (iii) (α_0_ = 0.95; β = 45). Typical time series of model behavior (the choice probability, learning rate, and actual choices) are described in Figure 1B. When the Q-learning model executes the task with a mistuned parameter, such as region (i) (α_0_ = 0.15; β = 45) and because the learning rate takes a constant value in the Q-learning model, α is the same as α_0_ and remains at a low value during the entire trial. Thus, the model cannot efficiently shift its action according to the situation, which leads to poor performance in the task. In another bad-parameter case, such as region (iii) (α_0_ = 0.95; β = 45), the model shifts its preference excessively, depending almost solely on the last trial even without changes in contingency, leading to poor performance. In contrast, the hybrid model can improve these situations by appropriately modulating the learning rate.

As shown in Figure 1B, the degree of learning rate changes increased with increases in the parameter η (in the range from 0 to 0.7), and the model's performance was improved, particularly in a mistuned parameter situation [e.g., regions (i) and (iii)]. This situation occurred because the model can detect the reversal of the good option more easily as the η value increased. Because η is a parameter that captures the level of influence of previous trials to the current learning rate, even if the model starts the task with a bad situation, the situation is improved according to the degree of past prediction error with an appropriate η value that leads gradually to an optimal action. However, if η is too large, such as η = 1, the action selection appears to be random, thereby leading to poor performance. When η = 1, the learning rate is similar to the absolute value of the prediction error of the previous trial, thus producing unstable actions.

**Figure 2:**
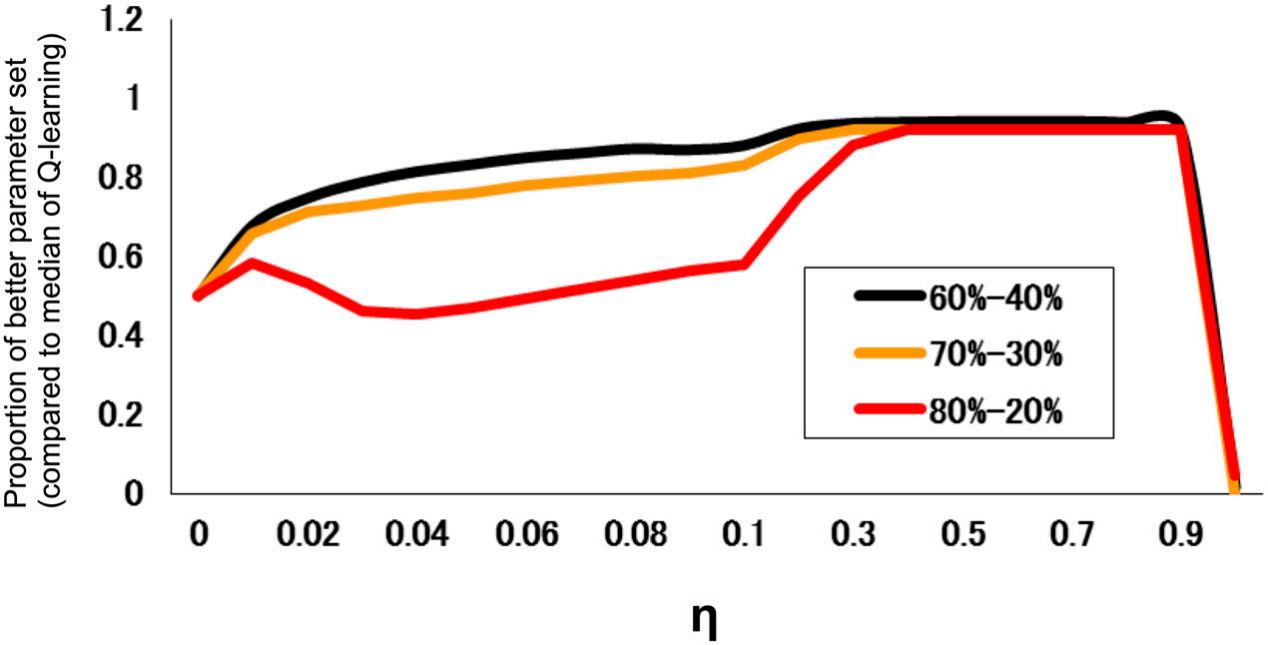
**Proportion of parameter regions in which the performance exceeds the median of the standard Q-learning for different task difficulties**. The proportion is defined as the fraction of the parameter set of α_0_ and β with a rate of advantageous choice that is larger than the median (across parameter combinations of α_0_ and β) of that of the standard *Q* learning rate (η = 0). The task difficulty is a measure of how difficult it is to distinguish a good choice from a bad choice; in this case, it took three levels (reward/loss frequency ratios of 80:20, 70:30, and 60:40; easier tasks have a higher ratio).

We also investigated the performance of models for different degrees of difficulty. Figure 2 indicates the proportion of parameter regions whose performance exceeds the median (across an entire parameter set) of the standard Q-learning for different task difficulty. The results indicated that the hybrid model did not necessarily outperform the standard Q-learning model at the low degree of difficulty (80–20%); however, the hybrid model outperformed the standard Q-learning model at a high degree of difficulty (60–40%).

## Model fitting in the reversal learning task

We conducted a statistical model fit to human behavioral data from the same task that was used in the computer simulation. The aim of the model fitting was to investigate whether the dual learning signal, value update, and learning rate modulation modeled by the hybrid model can explain human decision-making behavior more accurately than the standard Q-learning model.

### Methods

#### Participants

Fifteen healthy normal participants participated in this study (all of the participants were right handed; *n* = 6 females; age range = 20–25 years, mean = 21.2 years). The participants were preassessed to exclude those with a previous history of neurological or psychiatric illness. All of the participants provided informed consent after the nature of the study had been explained. All of the experimental procedures were approved by the Institutional Review Board of the Department of Psychology, Graduate School of Environmental Studies, Nagoya University.

#### Task description

The subjects participated in a simple probabilistic reversal learning task, in which the task structure was identical to the one that we had used for the computer simulation. Thus, the basic design was described above, and the specific focus of the experiment is described in this section. In each trial, the participants were presented with two abstract line drawings on the left and right sides of the screen (random left-right spatial position) and were requested to select one drawing. One stimulus, defined as the advantageous stimulus, led the participants to a monetary reward (winning 30 JPY) with a likelihood of 70% and a monetary loss (losing 30 JPY) with a likelihood of 30%; thus, choosing the advantageous stimulus led to a cumulative monetary gain. The other stimulus, which was defined as the disadvantageous stimulus, led the participants to a reward with a likelihood of 30% and to punishment with a likelihood of 70%. Thus, the disadvantageous stimulus led to a cumulative monetary loss. The task was constituted using 5 blocks.

Each block consisted of 160 trials. After 80 trials of a block, the contingencies were reversed. Once the reversal occurred, the participants had to choose a new advantageous stimulus. A different stimulus was used in each block; thus, the participants had to start learning from scratch in every block. The paradigm used here is based on that used in previous studies of probabilistic reversal learning (Ohira et al., 2011). The participants were advised that the task was a gamble for each trial, and they were provided no explicit instructions for the reversal of stimuli and reward/punishment. Additionally, the participants were told that money paid for experimental participation would be adjusted according to the gambling performance. The EEG data were also recorded during the task; however, we did not focus on the brain activity in the present study. Thus, we only reported the results using the behavioral data.

At the end of the experimental session, the participants were fully informed concerning the purpose of the experiment and were thanked for their participation and performance. Although the participants were told that they would receive money according to their performance, all of the participants were paid 1000 JPY for their participation.

#### Behavioral index

The response bias was defined as the rate of selection of the advantageous stimulus during the first and last 80 trials of the task, which is before and after the reversal occurred, respectively. The response bias was calculated for each participant.

#### Parameter fitting

We adopted the maximum-likelihood approach to fit the model parameters to the participants' choice behaviors. To exclude the effect of learning the task structure over blocks, only the date from the first block (160 trials) was used for the model-based analysis. If the participant's choice for the *t*th trial is (*t*), the likelihood is given by *P*(a(*t*)), which is computed from the soft-max function shown in Equation 2. The log-likelihood for the entire trial is as follows:

(5) $$L=\sum_{t\;=\;1}^{T}InP(\text{a}(t)),$$

where *T* denotes the total number of trials, which was 160 in our experiment. We computed this log-likelihood by initializing the *Q*-values at zero and updating the *Q*-values based on the actual participant's choice data. The fmincon function in MATLAB was employed to find the parameter set that produced the highest log-likelihood. To compare the goodness-of-fit of the two models with the best-fit parameters, we computed Akaike's information criterion (AIC), as shown in the following equation:

(6) AIC=−2L+2k,

where k is the number of free parameters (two for the standard Q-learning model, three for the hybrid model). The smaller AIC values indicated a better model. We also computed another model selection criterion, the Bayesian information criterion (BIC), which is shown in the following equation:

(7) BIC=−2logL+klogN,

where *N* is the total number of data points (Schwarz, 1978). These measures consider the number of free parameters in each model, which includes a penalty that increases as a function of the number of adjustable parameters, as shown in Equations 6 and 7.

### Results

#### Behavioral index

The response biases were 70 ± 4% before reversal occurred (mean ± s.e.m. for participants) and 61 ± 4% for after reversal occurred. No significant difference was observed between the first and last 80 trials.

#### Model-based analysis of choice behavior

We compared the goodness-of-fit of the standard Q-learning model and hybrid model. A single parameter set was estimated for all of the participants to obtain a stable estimator (Daw, 2011). The hybrid model is more complex than the standard Q-learning model, and the standard Q-learning model is a special case of the hybrid model, i.e., the standard Q-learning model can be represented by setting η = 0 in the hybrid model. First, we used the classical likelihood ratio tests of the null hypothesis that the improvement in fit of the more complicated model (the hybrid model) relative to the simpler model (the standard Q-learning model) was expected by chance. The likelihood ratio test is a statistical test that is used to compare the fit of two models, one of which (the null model) is a special case of the other (the alternative model). For example, in this study, the standard Q-learning model (the null model) is nested in the hybrid model (the alternative model) by setting η = 0. The two competing models were separately fitted to the data, and the log-likelihood was recorded. The test statistic is twice the difference in these log-likelihoods.

**Figure 3:**
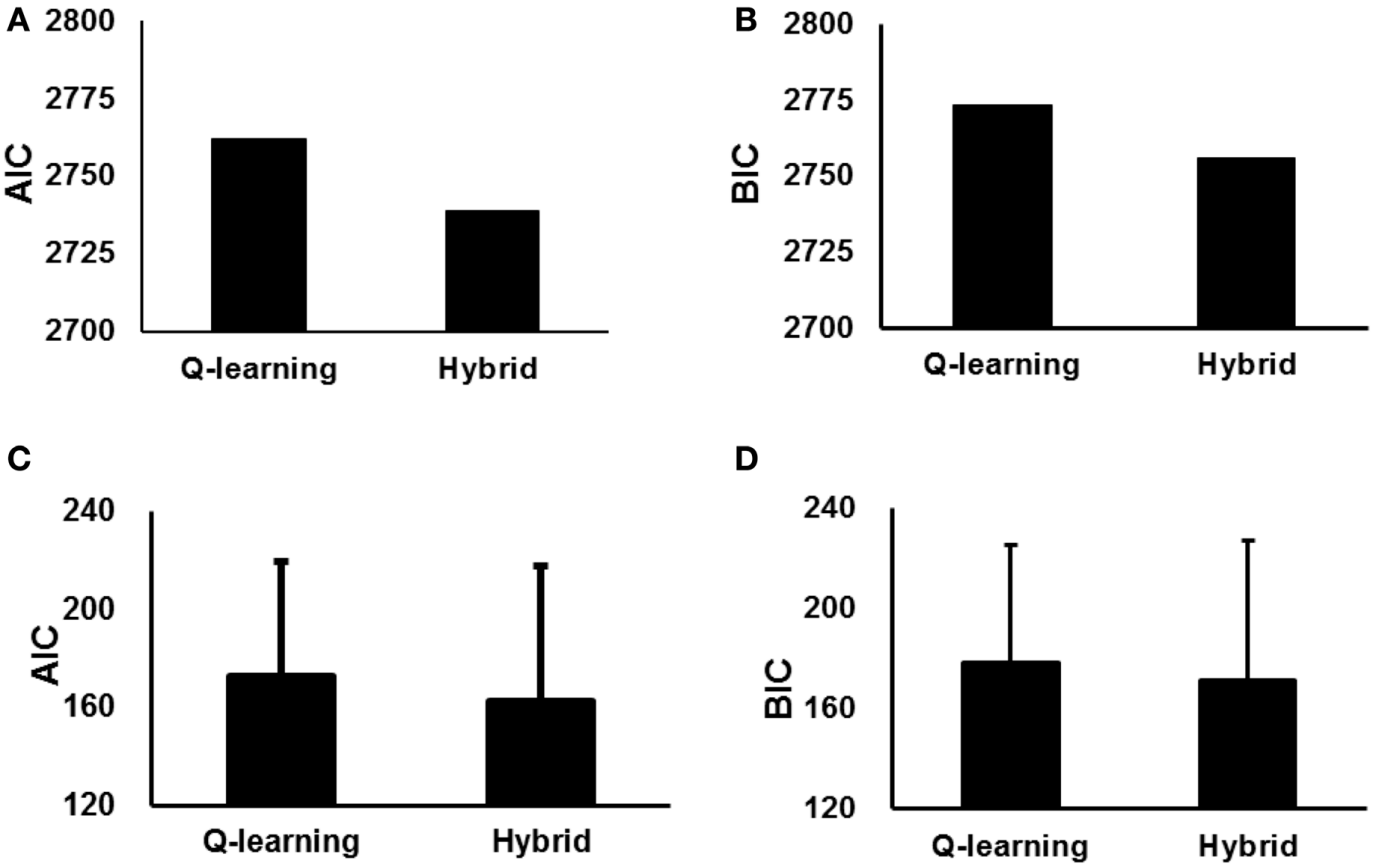
**Goodness-of-fit of the standard Q-learning model and hybrid model with parameters fit to the entire group (A,B), with parameters fit to individual participants (C,D). (A,C)** AIC scores of the two models. **(B,D)** BIC scores of the two models. The error bar indicates SD.

We also used the AIC and BIC to measure the goodness-of-fit. The parameter estimates and AIC and BIC measures for the standard Q-learning model and hybrid model are summarized in Table 1. The hybrid model yielded a significantly better fit than the standard Q-learning model at the likelihood ratio test [χ_(1)_ = 47.8; *p* < 0.001]. Additionally, the hybrid model exhibited a better fit than the standard Q-learning model when considering the AIC and BIC results (Table 1, Figure 3).

**Table 1:** **Model fit results (group fit)**.

**Model**	**LL**	**AIC**	**BIC**	**α(α_0_)**	**β**	**η**
Q-learning	−1379	2762	2773	0.114	2.584	–
Hybrid	−1366	2739	2756	0.018	2.601	0.004

*Shown are negative log-likelihood (−LL), AIC, BIC, and maximum-like maximum-likelihood parameter estimates for the standard Q-learning model and hybrid model with parameters fit to the entire group*.

We have not only estimated a single parameter set for the participants as a whole but also fit these models separately to each individual participant's behavior and individually performed likelihood ratio tests on the data likelihoods (Table 2, Figure 3). The results indicated that the hybrid model fit better than the standard Q-learning model for 9/15 participants at *p* < 0.05 and exhibited such a trend at *p* < 0.1 for an additional participant. The AIC and BIC were also computed for each individual participant. The hybrid model was significantly better than the standard Q-learning model in terms of the AIC [paired-sample *t*-test, *t*_(15)_ = 2.06, *p* < 0.05]. The BIC results indicated that the hybrid model was better than the standard Q-learning model [paired-sample *t*-test, *t*_(15)_ = 1.81, *p* < 0.1].

**Table 2:** **Model fit results (individual fit)**.

**Model**	**−LL**	**AIC**	**BIC**	**α (α_0_)**	**β**	**η**
Q-learning	−84 ± 23	173 ± 47	179 ± 47	0.08 ± 0.04	6.98 ± 12.12	–
Hybrid	−78 ± 28	162 ± 56	172 ± 56	0.18 ± 0.35	7.2 ± 12.18	0.07 ± 0.12

*Shown are negative log-likelihood (−LL), AIC, BIC, and maximum-like maximum-likelihood parameter estimates for the standard Q-learning model and hybrid model with parameters fit to the individual subjects*.

Notably, however, in the group fit results, α (α0) is much larger in the Q-learning model than in the hybrid model. This situation is reversed in the individual fit results. The following reasons may explain this difference. First, because parameter α(α_0_ for the hybrid model) is bounded within the range [0,1] and the estimated values were near zero, the variance among individuals forces the estimated value to have a bias toward positive large values, i.e., the greater the individual variance, the greater the population mean of the estimated value. Additionally, as the standard deviation of α_0_ forthe hybrid model was larger than that for standard Q-learning, the hybrid model may have a larger value of α_0_ for individual fit due to this bias.

## Discussion

In the present study, we investigated the functional significance of the hybrid learning algorithm using computer simulations with a reversal learning task. We also addressed the question as to whether features that are described by the hybrid model can be confirmed by actual human behaviors using the statistical model fit for human behavioral data.

The computer simulation results indicated that an agent cannot adjust its behavior effectively based on the standard Q-learning model when the agent starts the task with several inappropriate parameters (e.g., region (i), in which the decision maker cannot efficiently shift his/her action according to the situation due to the low learning rate, and region (iii), in which the decision maker shifts his/her preference excessively due to the high learning rate when η = 0, as shown in Figure 1). These situations can be improved in the hybrid model by updating its learning rate. Of interest is how the hybrid model can modify the performance when an agent starts the task using inappropriate parameters, such as regions (i) and (iii) in the learning process. As we mentioned previously, different types of conflicting abilities are required to perform this task effectively: (i) the ability of option discrimination and (ii) the ability to choose the good option consistently, even when occasionally receiving punishment. The computer simulation results suggest that the standard Q-learning model with a constant and large learning rate updates the value function considerably, which enables the decision maker to discriminate the difference of rewarding probabilities between the efficiency of options but makes it impossible to maintain the good option. If a decision maker has a small learning rate with the standard Q-learning model, the value function will be updated slightly, and the small learning rate enables the decision maker to obstinately maintain an option while being aware that switching to the good option becomes more difficult over time. Thus, the decision maker faces a trade-off between these requirements. The learning rate parameter determines the balance between these two functions. Thus, the standard Q-learning model is vulnerable to bad tuning of the initial parameters. In contrast, the hybrid model utilizes the prediction error to control the learning rate and can hence more flexibly update the value function; even the initial learning rate was maintained at some extreme value. Therefore, the behavior based on this process is perceived to be more robust against the mistuning of parameters and can work in a wider field of parameters than the standard Q-learning model.

In the present study, we compared the two models at three different degrees of task difficulty and defined the difficulty by the similarity in reward probability between two options. A smaller difference indicates that it is more difficult to reconcile the trade-off. For example, in a difficult condition (reward/loss frequency ratio of 60:40), there is not a considerable difference between a good and bad option in terms of reward contingency, and decision makers will frequently encounter a loss even when they only choose the good option. Therefore, when the decision makers are placed in a volatile environment, if the learning rate is set at a high constant value, then the higher updating of decision value will be frequently increased by punishment, making the behavior unstable. If the learning rate is set at a small constant value, then the discrimination will become more difficult. Hence, the superior performance of the hybrid model may become more pronounced in a difficult condition. In accordance with this speculation, the present results indicated that the hybrid model outperformed the standard Q-learning model more significantly for a task with a high degree of difficulty (60–40%) compared to a task with a low degree of difficulty (80–20%) (Figure 2). These simulation results reveal that the hybrid model is preferable over the standard Q-learning model, especially in an environment in which it is difficult to discriminate a good option.

Additionally, the statistical model fit data indicated that the hybrid model has more explanatory power than the Q-learning model for the behavioral data. However, the estimated value of parameters is not the same with the optimal parameter sets obtained by the computer simulations. Thus, the participants' performance was not necessarily based on an optimal hybrid process. Nevertheless, the results of the model comparison suggest the existence of a dual learning mechanism involving the value update and learning rate modulation that are described by the hybrid model in the human's action selection.

In a previous study, Erdeniz and Atalay (2010) used a computer simulation to investigate the performance of a neural network model called the attention-gated reinforcement learning (AGREL) model in a probabilistic reversal learning task. The AGREL model is an algorithm that includes two processes: (1) a feed-forward process that relates the effect of unexpected outcomes on learning and (2) a feedback attention process that relates the effect of top-down attention on updating weights. These processes respectively correspond to the processes of value update based on reward prediction error and learning rate modulation based on the surprise signal, which is described in the hybrid model. Thus, the hybrid model could be expected to have an effect similar to that in the ARGEL algorithm when using a probabilistic learning task and a reversal learning task. Although Erdeniz and Atalay (2010) showed that the AGREL model can quickly adapt to the change in reinforcement contingencies, they did not systematically investigate the influence of parameter tuning. The present study showed the robustness against the mis-tuning of parameter settings in the hybrid model. More specifically, we found that the standard RL model can also adapt the dynamic change in the reinforcement contingencies within a certain range of parameter settings. However, the hybrid model yields comparable performance for a broad range of parameter settings to that of the standard RL model. Furthermore, Erdeniz and Atalay (2010) indicated that a possible beneficial effect of the utility of the AGREL model for fitting behavior data is required in future experiments. As mentioned above, in the present study, the statistical model fit data suggested that the hybrid model has more explanatory power than the Q-learning model for the behavioral data.

The physiological correspondence of the hybrid model in the action selection task remains unclear. Li et al. (2011) indicated that BOLD responses in the bilateral amygdala correlated with the learning rate in the hybrid model and that autonomic reactivity, such as skin conductance responses (SCRs), might directly reflect learning rate modulation in a Pavlovian reversal-learning task. One interesting future direction is to investigate whether the relationship between the associability and physiological activities suggested by Li et al. (2011) can also be observed in an action selection task using a model-based analysis, as we conducted in the present study.

Furthermore, in the present study, we used a reversal learning task in which the reversal occurred only once. Whereas this task design was simple and has been adopted in many studies (Butter, 1968; Rolls et al., 1994; McAlonan and Brown, 2003; Tsuchida et al., 2010), the extent to which the functional role of the hybrid model, which was revealed in the present study, can be applied to the general learning process remains unclear. This functional role may depend on the frequency of reversal and the type of reinforcement schedule: the factors can modulate the volatility of circumstances. A previous work (Behrens et al., 2007) reported that the learning rate of human decision makers became higher in a volatile condition than in a stable condition. A challenge for the future is to clarify the general function of the hybrid model in different tasks, including those in which the reward probabilities follow a random walk method or those in which the reward magnitude varies along with the reward probability in which the expected reward must be learned.

### Conflict of interest statement

The authors declare that the research was conducted in the absence of any commercial or financial relationships that could be construed as a potential conflict of interest.
